# lncRNA NEAT1 mediates sepsis progression by regulating Irak2 via sponging miR-370-3p

**DOI:** 10.1242/bio.049353

**Published:** 2020-06-24

**Authors:** Ting Xiao, Chuihua Sun, Ying Xiao, Yunbao Li

**Affiliations:** 1 Department of Infectious Diseases, Weifang People's Hospital, Weifang, Shandong, 261041, China; 2 Department of Intensive Care Unit, Weifang People's Hospital, Weifang, Shandong, 261041, China; 3 Department of Ultrasound, 960 Hospital of the Chinese People's Liberation Army (Tai'an Hospital), Tai'an, Shandong, 271000, China; 4 Department of Clinical Laboratory, Jinan Chain Medical Laboratory Co., Ltd, Jinan, Shandong, 250000, China

**Keywords:** Sepsis, NEAT1, miR-370-3p, Irak2

## Abstract

Sepsis is a life-threatening condition and often associated with multiple organ failure. Nuclear-enriched abundant transcript 1 (NEAT1), a member of the long non-coding RNAs (lncRNAs), was reported to be involved in the regulation of sepsis progression. However, its precise regulatory mechanism needs to be further explored. In this study, the cell-counting kit-8 assay was used to check cell viability. The quantitative real-time polymerase chain reaction (qRT-PCR) was employed to detect the expression levels of NEAT1, miR-370-3p and Interleukin 1 receptor associated kinase 2 (Irak2). Flow cytometry assay and ELISA were used to check cell apoptosis and the concentrations of inflammatory cytokines, respectively. The starBase was used to predict binding sites between miR-370-3p and NEAT1 or Irak2 and the dual-luciferase reporter assay was performed to verify the interaction. The protein level of Irak2 in samples was measured by western blot. The high concentration of lipopolysaccharide (LPS) led to the high death ratio of RAW 264.7 and HL-1 cells. NEAT1 and Irak2 were upregulated in sepsis tissues and LPS-induced RAW 264.7 and HL-1 cells, opposite to the expression of miR-370-3p. In addition, knockdown of NEAT1 promoted viability, suppressed apoptosis and reduced the expression of inflammatory cytokines in LPS-induced RAW 264.7 and HL-1 cells. Moreover, we found that miR-370-3p interacted with NEAT1 and targeted the 3′UTR of Irak2. Further research indicated that downregulation of miR-370-3p or upregulation of Irak2 rescued NEAT1 silencing-mediated inhibitory effect on sepsis progression. Knockdown of NEAT1 hampered sepsis progression by downregulating Irak2 via interacting with miR-370-3p in LPS-induced RAW 264.7 and HL-1 cells.

## Abstract (precis)

**Summary:** Knockdown of NEAT1 hampered sepsis progression by downregulating Irak2 via interacting with miR-370-3p in LPS-induced RAW 264.7 and HL-1 cells.

## INTRODUCTION

Sepsis is caused by the inflammatory immune responses triggered by an infection and is a leading cause of morbidity and mortality worldwide (Deutschman and Tracey, 2014). The risk of death from sepsis ranges from about 50% (severe sepsis) to nearly 80% (septic shock) (Jawad et al., 2012). Therefore, it is imperative to figure out the pathogenesis of sepsis for its future prevention and treatment.

Long noncoding RNAs (lncRNAs) are a type of RNA molecule (more than 200 nucleotides) that lose the ability to encode proteins (Mercer et al., 2009). lncRNAs were reported to participate in the modulation of sepsis (Chen et al., 2019; Wang et al., 2018, 2019b). Previous reports demonstrated that lncRNA nuclear-enriched abundant transcript 1 (NEAT1) was involved in the development of diverse human cancers, including myeloma (Taiana et al., 2019), breast cancer (Li et al., 2019a) and cervical cancer (Yuan et al., 2019). Recently, NEAT1 was reported to be correlated with the progression of inflammation-related diseases (Liu et al., 2019; Wang et al., 2019a; Zhang et al., 2019). Though these studies showed that NEAT1 was closely associated with sepsis-induced injury, the regulatory mechanism of NEAT1 in sepsis progression is still worth studying.

MicroRNAs (miRNAs) are short (about 22 nucleotides) noncoding RNAs, which mediate gene expression via guiding Argonaute proteins to target sites in the 3′-untranslated region (3′UTR) of messenger RNA (mRNA) (Gebert and MacRae, 2019). Growing evidence has shed light on the fact that miRNAs function in the regulation of sepsis progression (Lin et al., 2019; Ling et al., 2019; Shen et al., 2019). Recent research has shown that miR-370-3p regulated inflammation injury in acute pneumonia (Zhang et al., 2019). Nevertheless, the potential mechanism of miR-370-3p in sepsis progression needs to be further explored.

Interleukin 1 receptor associated kinase 2 (Irak2) was involved in many human cancers. Liu et al. found that Irak2 counterbalanced oncogenic smurf1 in colon cancer cells (Liu et al., 2018). Xu et al. reported that Irak2 could be a predictor of non-small lung cancer (Xu et al., 2018). A recent report demonstrated that Irak2 was crucial for lipopolysaccharide (LPS)-mediated post-transcriptional control (Wan et al., 2009). Therefore, Irak2 may be an attractive drug target for sepsis and new regulators regulating Irak2 need to be determined.

In this research, the expression level of NEAT1 in sepsis tissues and LPS-induced RAW 264.7 and HL-1 cells was checked. The function and underlying regulatory mechanism of NEAT1 in sepsis were further investigated by subsequent experiments.

## RESULTS

### LPS inhibited the viability of RAW 264.7 and HL-1 cells

**Fig. 1.**
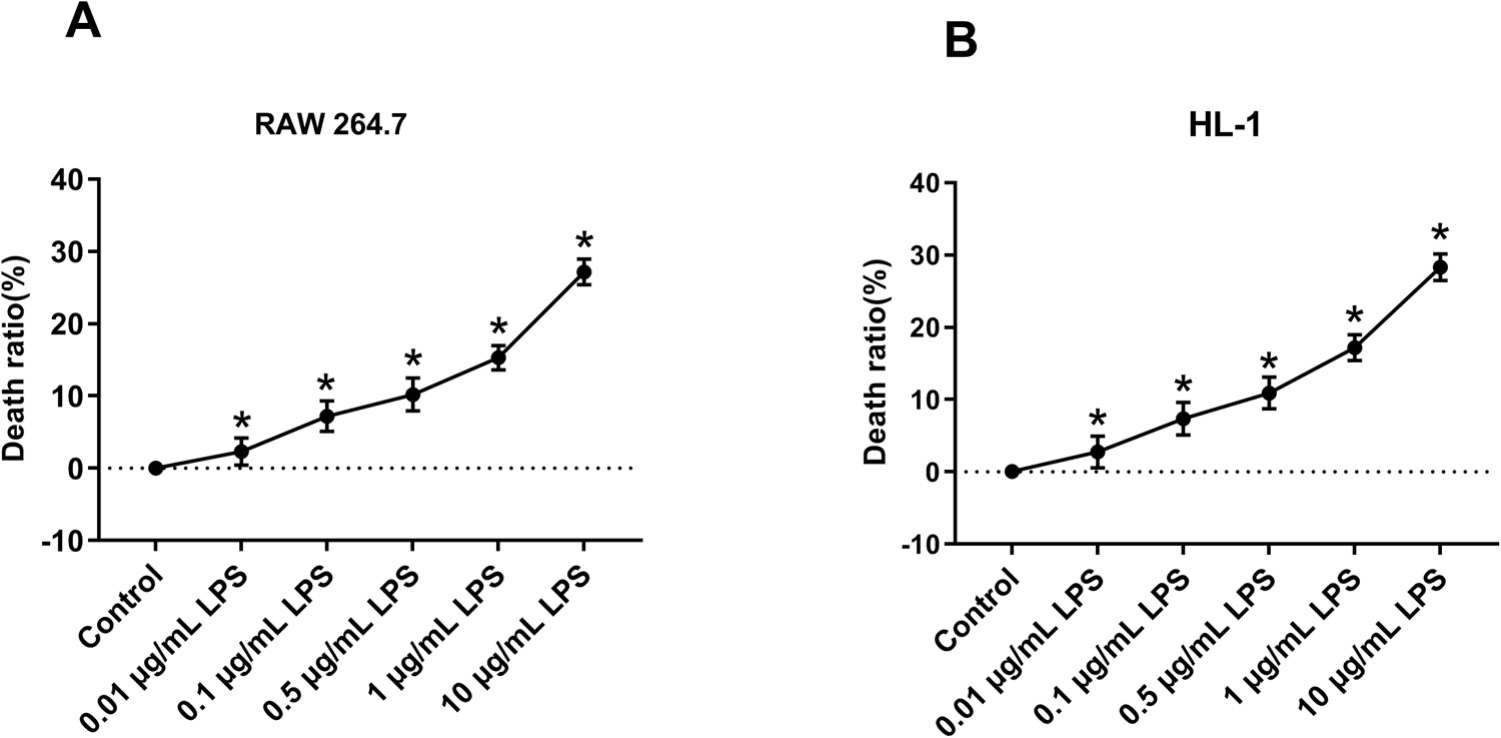
**High concentration of LPS led to high death ratio of RAW 264.7 and HL-1 cells.** (A,B) CCK-8 assay was used to check the effect of LPS (0, 0.01, 0.1, 0.5, 1 and 10 μg/ml) on RAW 264.7 and HL-1 cells. *P<0.05.

As a main pathogenic factor of sepsis, LPS could trigger the inflammatory cascade, inducing necrosis and apoptosis of epithelial cells (Li et al., 2018). In this research, RAW 264.7 and HL-1 cells were treated with LPS in different concentrations and the cell counting kit-8 (CCK8) assay was used to check cell viability. The result showed that the cell-death ratio was gradually increased with the increasing LPS concentrations (Fig. 1A,B) and 1 μg/ml LPS was chosen for subsequent experiments. These results indicated that LPS contributed to the high cell-death ratio in a dose-dependent manner.

### NEAT1 was upregulated in sepsis tissues and LPS-induced RAW 264.7 and HL-1 cells

**Fig. 2.**
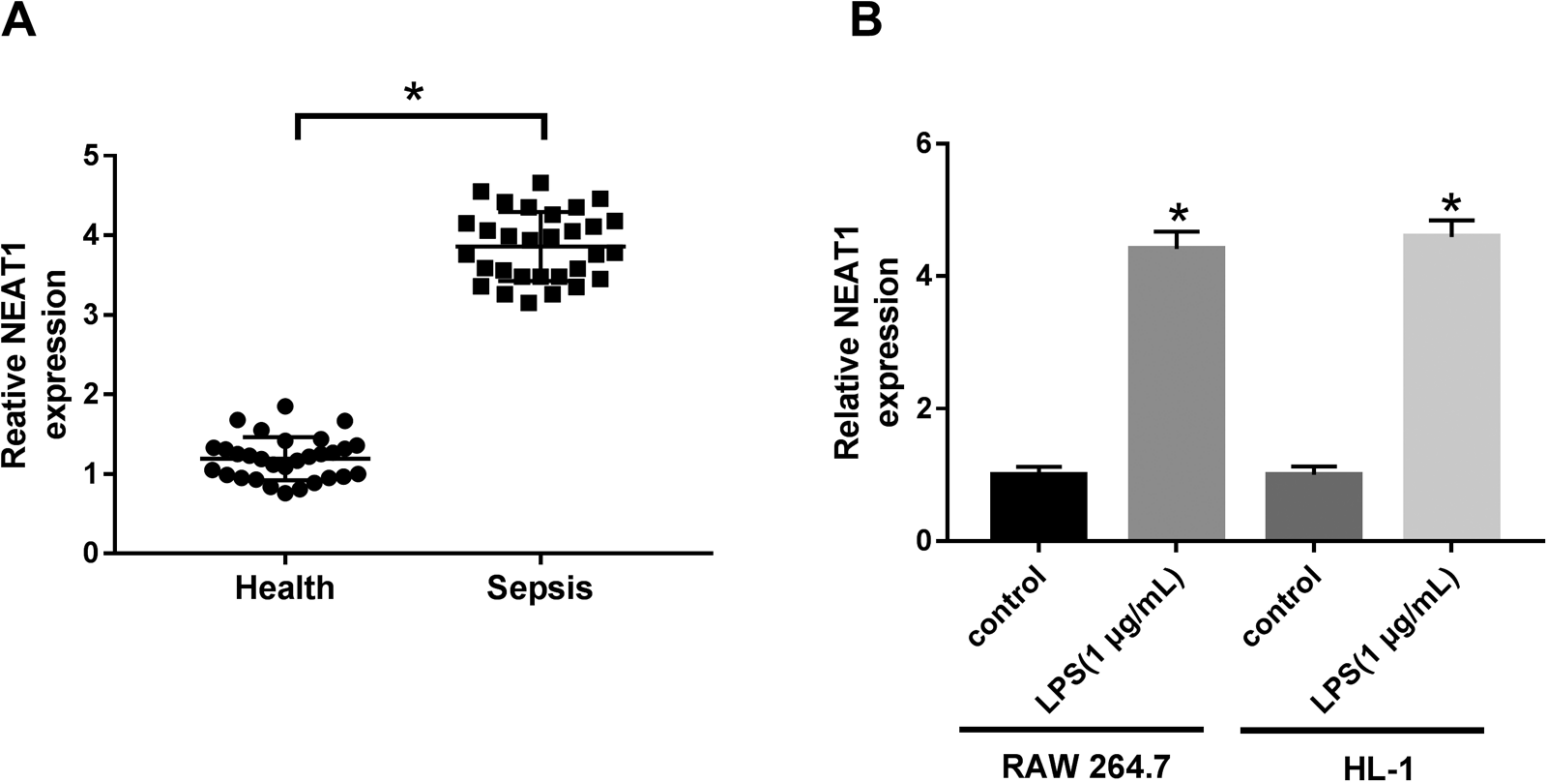
**The level of NEAT1 was increased in sepsis tissues and LPS-induced cells.** (A,B) The expression of NEAT1 in sepsis tissues and LPS-induced RAW 264.7 and HL-1 cells, as well as matched controls, was checked by qRT-PCR. *P<0.05.

To explore the role of NEAT1 in sepsis, first, its expression was measured and the results showed that NEAT1 was obviously upregulated in sepsis tissues compared with healthy tissues (Fig. 2A). Similarly, the level of NEAT1 was significantly increased in LPS-induced RAW 264.7 and HL-1 cells compared with corresponding controls (Fig. 2B). From these results, it could be concluded that NEAT1 might be a vital immunoregulatory factor and have a diagnostic value in sepsis.

### Downregulation of NEAT1 promoted viability and inhibited apoptosis and inflammatory cytokine secretion in LPS-induced RAW 264.7 and HL-1 cells

**Fig. 3.**
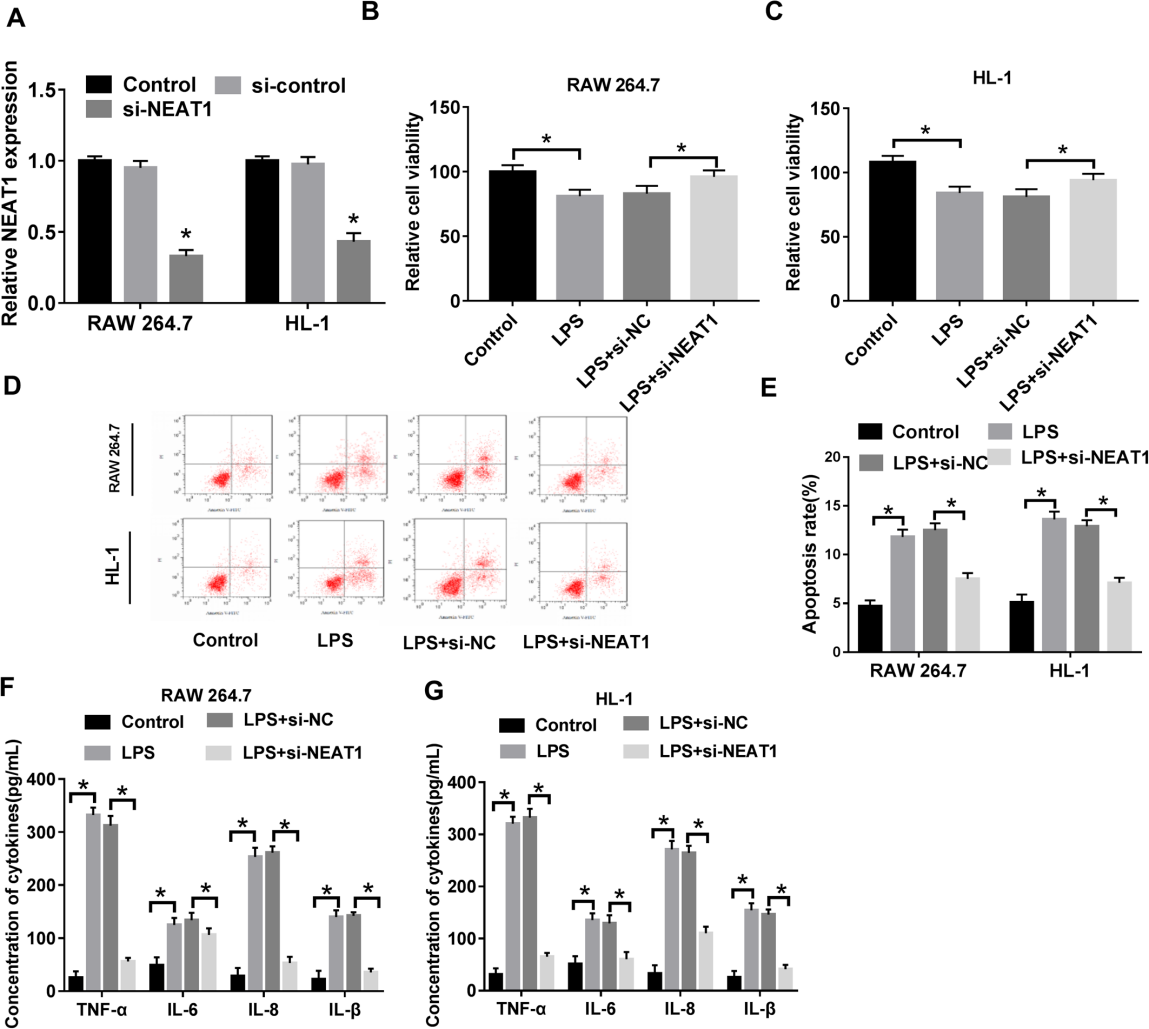
**NEAT1 silencing retarded sepsis progression.** (A,B) The level of NEAT1 in LPS-induced cells infected with si-NEAT1 and corresponding controls was measured by qRT-PCR. (C) Cell viability was checked by CCK-8 assay. (D,E) Flow cytometry was employed to detect cell apoptosis. (F,G) The concentrations of TNF-α, IL-6, IL-8 and IL-β in samples were checked by ELISA assay. *P<0.05.

To investigate the function of NEAT1 in sepsis progression, we first checked the level of NEAT1 in LPS-induced RAW 264.7 and HL-1 cells infected with small interfering (si)-NEAT1 and matched controls. The data showed that NEAT1 was markedly decreased in si-NEAT group (Fig. 3A). Afterwards, CCK8 assay was performed and the data showed that knockdown of NEAT1 clearly promoted the viability of LPS-induced RAW 264.7 and HL-1 cells (Fig. 3B,C). In addition, a flow cytometry assay indicated that downregulation of NEAT1 conspicuously suppressed the apoptosis of LPS-induced cells (Fig. 3D,E). Moreover, the concentrations of inflammatory cytokines were measured and the results disclosed that NEAT1 silencing strikingly decreased the levels of tumor necrosis factor (TNF)-α, interleukin (IL)-6, IL-8 and IL-β in LPS-induced RAW 264.7 and HL-1 cells (Fig. 3F,G). Collectively, these results demonstrate that silencing NEAT1 hindered the progression of sepsis.

### NEAT1 targeted and negatively regulated miR-370-3p in LPS-induced RAW 264.7 and HL-1 cells

**Fig. 4.**
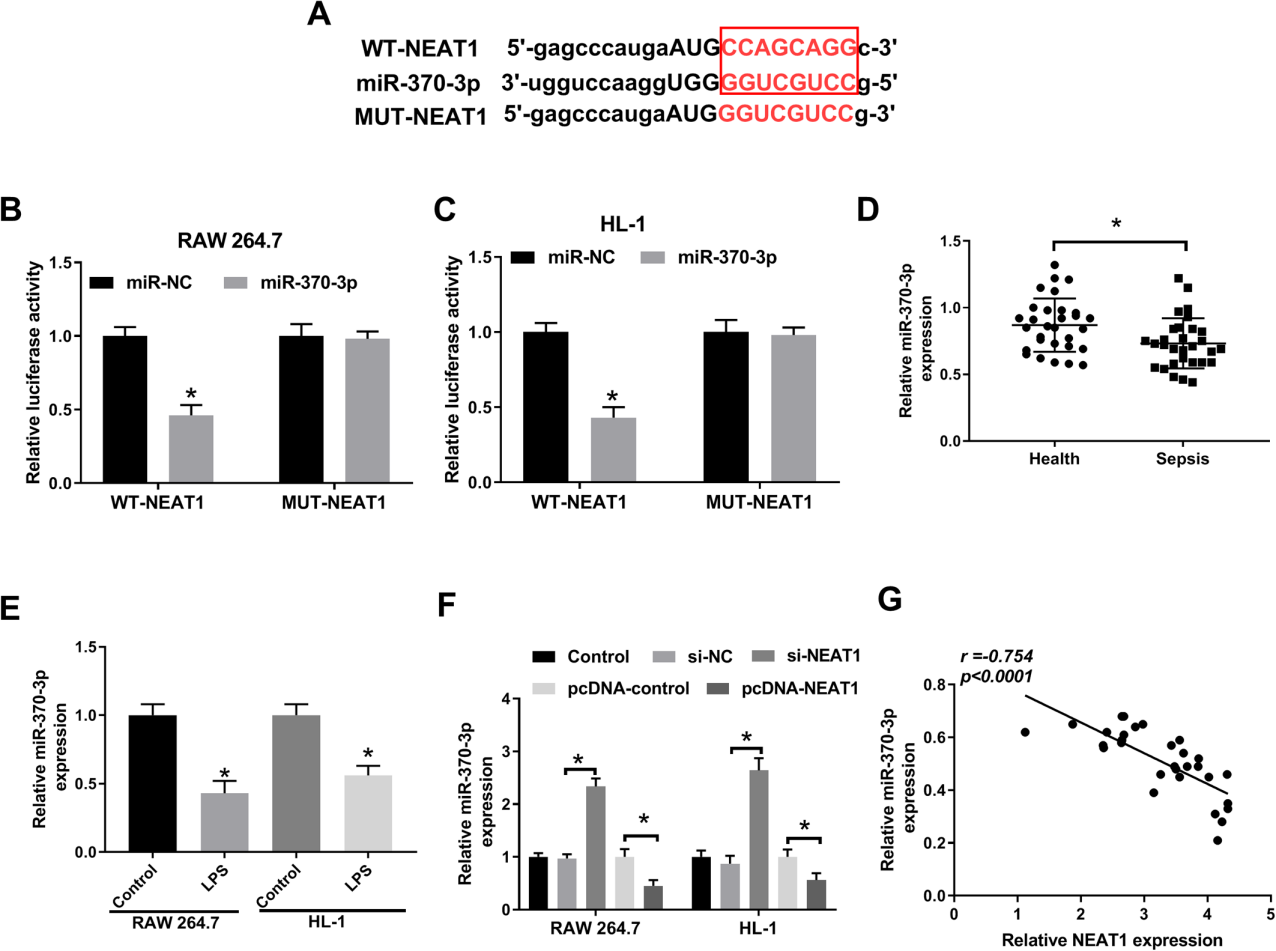
**NEAT1 interacted with and negatively regulated miR-370-3p.** (A) The possible binding sites between miR-370-3p and NEAT1 were predicted by starBase. (B,C) The dual-luciferase reporter assay was performed to verify the interaction between miR-370-3p and NEAT1. (D) The expression of miR-370-3p in sepsis tissues and healthy tissues was assessed by qRT-PCR. (E) The expression of miR-370-3p in RAW 264.7 and HL-1 cells treated with LPS or not was estimated by qRT-PCR. (F) The level of miR-370-3p in LPS-induced cells infected with si-NEAT1 or pcDNA-NEAT1, as well as matched controls, was evaluated by qRT-PCR. (G) The correlation between NEAT1 and miR-370-3p in sepsis tissues was analyzed using Pearson's correlation coefficient. *P<0.05.

Growing evidence has reported that lncRNAs could target miRNAs to regulate the progression of sepsis (Wang et al., 2018, 2019b). In this research, miR-370-3p was predicted to be a target of NEAT1 by starBase (Fig. 4A) and the dual-luciferase reporter assay indicated that miR-370-3p markedly reduced the luciferase activity of WT-NEAT1 in LPS-induced RAW 264.7 and HL-1 cells, rather than MUT-NEAT1 (Fig. 4B,C). Subsequently, the level of miR-370-3p was checked and the results indicated that miR-370-3p apparently decreased in sepsis tissues and LPS-induced cells (Fig. 4D,E). Further studies showed that downregulation of NEAT1 noticeably elevated the level of miR-370-3p, while overexpression of NEAT1 markedly reduced the expression of miR-370-3p (Fig. 4F). In addition, the expression of miR-370-3p correlated negatively with NEAT1 in sepsis tissues (Fig. 4G). Altogether, these results suggested that miR-370-3p was a target of NEAT1 and downregulated by NEAT1.

### Downregulation of miR-370-3p reversed NEAT1 silencing-mediated effects on viability, apoptosis and inflammatory cytokine secretion

**Fig. 5.**
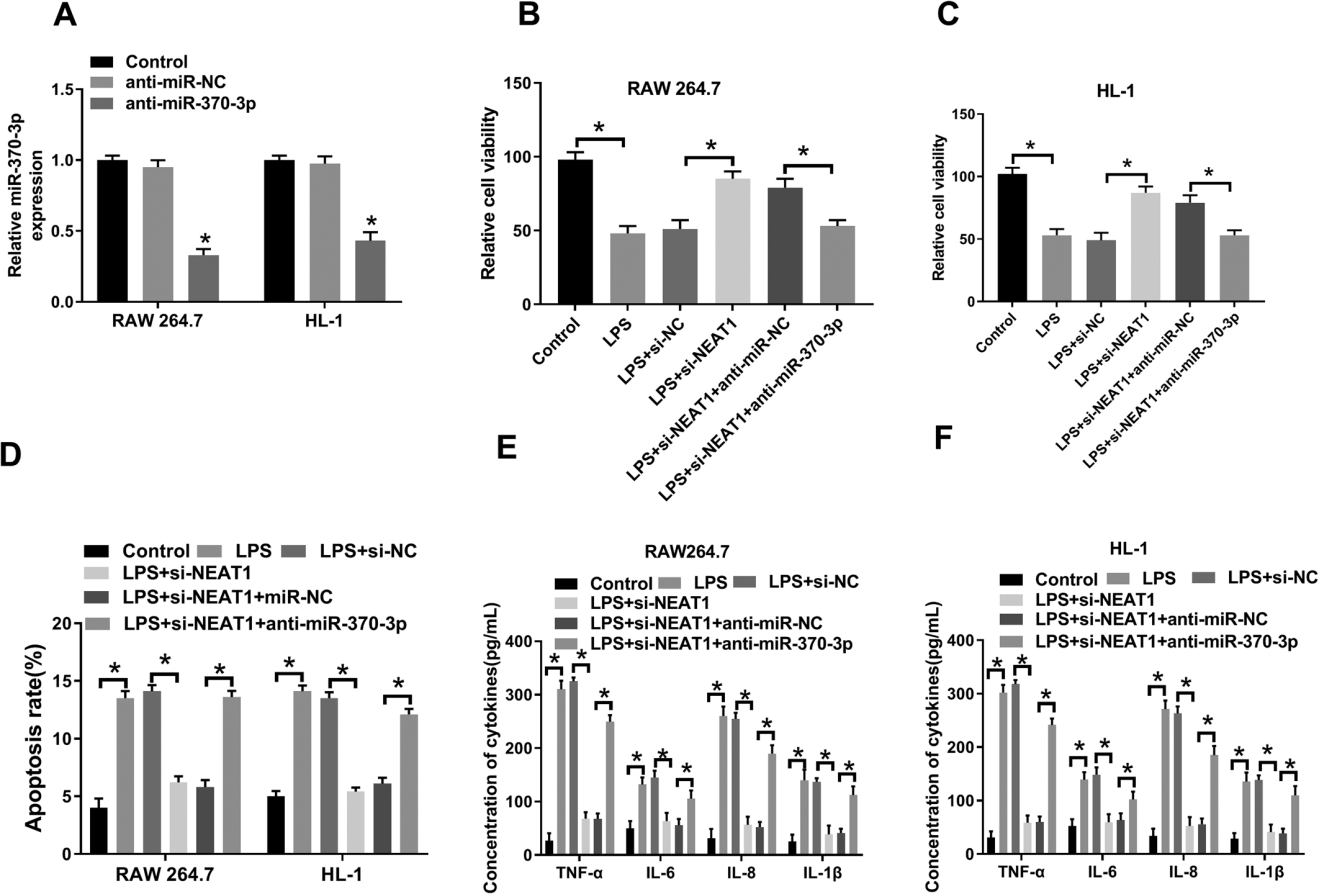
**MiR-370-3p depletion rescued NEAT1 silencing-mediated effect on sepsis progression.** (A) The level of miR-370-3p in LPS-induced cells infected with miR-370-3p mimic and corresponding controls was checked by qRT-PCR. (B,C) Cell viability was measured by CCK-8 assay. (D) Flow cytometry was hired to analyze cell apoptosis. (E,F) The concentrations of TNF-α, IL-6, IL-8 and IL-β in cells were checked by ELISA assay. *P<0.05.

To elucidate the potential regulatory mechanism of miR-370-3p and NEAT1 in sepsis progression, LPS-induced RAW 264.7 and HL-1 cells were first transfected with anti-miR-370-3 or anti-miR-NC and the knockdown efficiency was confirmed (Fig. 5A). Thereafter, cell viability was evaluated and the data indicated that the NEAT1 silencing effect on cell viability was reversed by downregulating miR-370-3p (Fig. 5B,C). Meanwhile, a flow cytometry assay showed that miR-370-3p depletion inverted the inhibitory effect of NEAT1 silencing on cell apoptosis (Fig. 5D). Moreover, the decreased levels of TNF-α, IL-6, IL-8 and IL-β in LPS+si-NEAT1 group were overturned following infection with anti-miR-370-3p (Fig. 5E,F). Together, these results demonstrated that NEAT1 and miR-370-3p played opposite roles in sepsis progression and NEAT1-regulated sepsis progression via interacting with miR-370-3p.

### MiR-370-3p targeted and negatively regulated Irak2 in LPS-induced RAW 264.7 and HL-1 cells

**Fig. 6.**
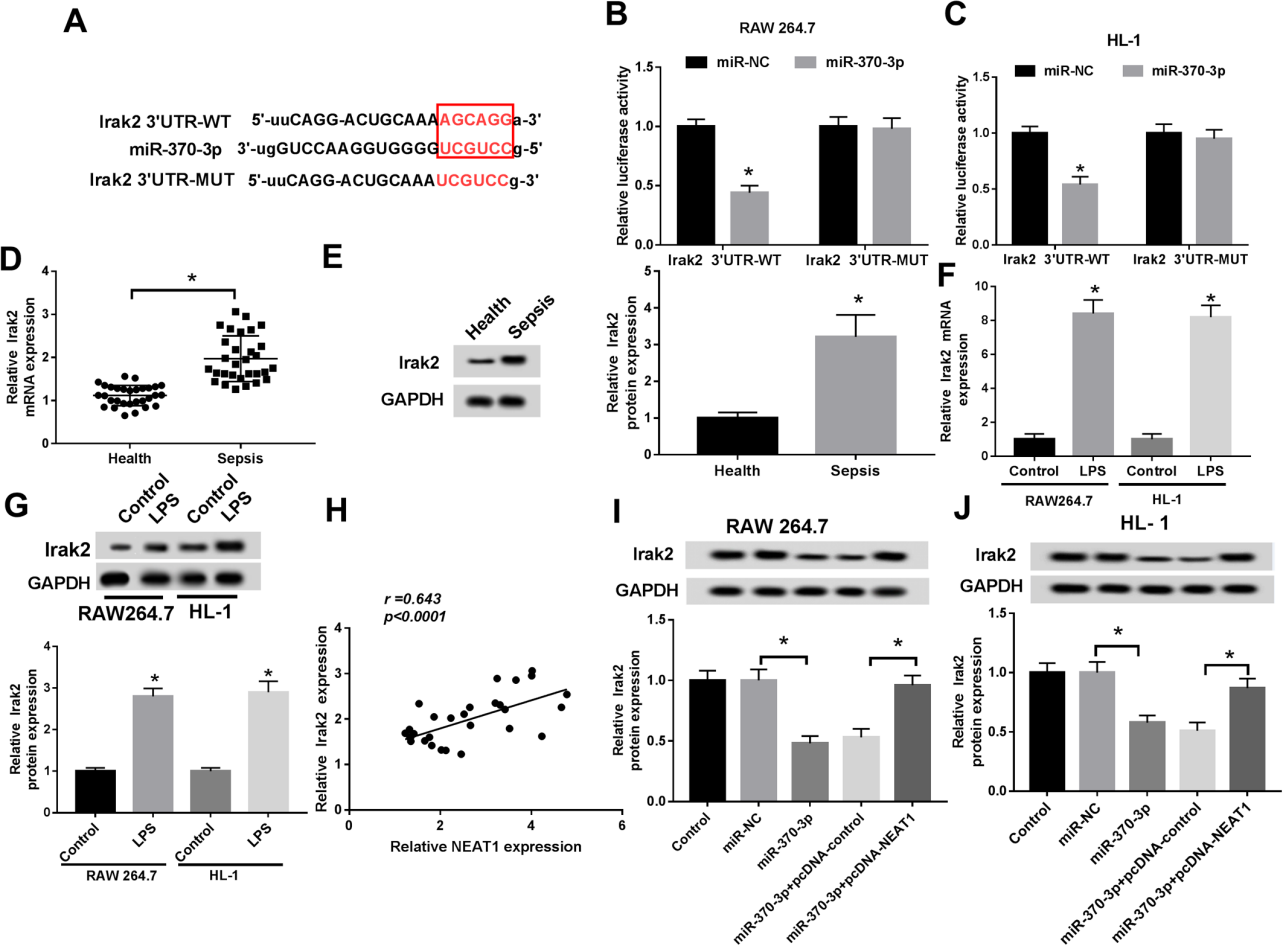
**MiR-370-3p targeted and negatively regulated Irak2.** (A) The potential target sites between miR-370-3p and Irak2 were forecasted by starBase. (B,C) The dual-luciferase reporter assay was carried out to confirm the interaction between miR-370-3p and Irak2. (D,E) The mRNA and protein levels of Irak2 in sepsis tissues and healthy tissues were checked by qRT-PCR and western blot, respectively. (F,G) The mRNA and protein levels of Irak2 in RAW 264.7 and HL-1 cells treated with LPS or not were measured by qRT-PCR and western blot, respectively. (H) The correlation between miR-370-3p and Irak2 in sepsis tissues was analyzed using Pearson's correlation coefficient. (I,J) The protein level of Irak2 in LPS-induced cells infected with miR-370-3p mimic or pcDNA-NEAT1, as well as matched controls, was detected by western blot. *P<0.05.

To further probe the regulatory mechanism of miR-370-3p, its possible target genes were predicted by starBase and the result showed that miR-370-3p could bind to the 3′UTR of Irak2 (Fig. 6A), which was corroborated by the dual-luciferase reporter assay (Fig. 6B,C). Next, we checked the expression of Irak2 and the data indicated that Irak2 was clearly increased in sepsis tissues (Fig. 6D,E) and LPS-induced RAW 264.7 and HL-1 cells (Fig. 6F,G). Correlation analysis showed that the expression of Irak2 was positively associated with NEAT1 in sepsis tissues (Fig. 6H). Furthermore, Irak2 was downregulated in miR-370-3p group, whereas its expression level was upregulated after the transfection with pcDNA-NEAT1 (Fig. 6I,J). To sum up, these results illuminated that NEAT1 modulated the expression of Irak2 by targeting miR-370-3p in LPS-induced RAW 264.7 and HL-1 cells.

### Overexpression of Irak2 inverted NEAT1 silencing-mediated impacts on viability, apoptosis and inflammatory cytokine secretion

**Fig. 7.**
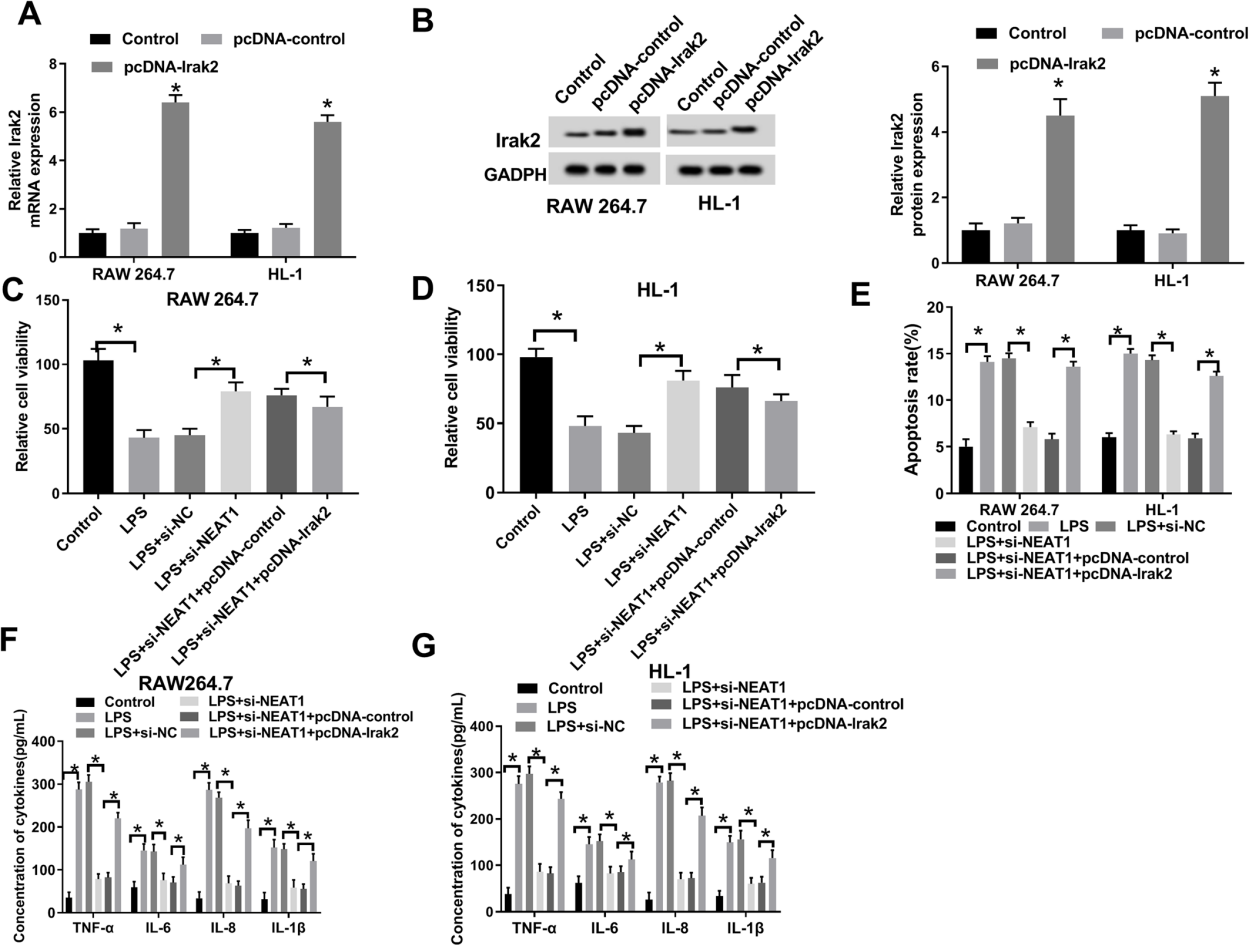
**NEAT1-regulated sepsis progression by miR-370-3p/Irak2 axis.** (A,B) The mRNA and protein levels of Irak2 in LPS-induced cells infected with pcDNA-Irak2 and corresponding controls were measured by qRT-PCR and western blot, respectively. (C,D) CCK-8 assay was used to check cell viability. (E) Flow cytometry was used to check cell apoptosis. (F,G) The concentrations of TNF-α, IL-6, IL-8 and IL-β in LPS-induced cells infected with si-NEAT1 or pcDNA-Irak2 and matched controls were detected by ELISA assay. *P<0.05.

To study the relationship between Irak2 and NEAT1 in sepsis progression, we first transfected LPS-induced RAW 264.7 and HL-1 cells using pcDNA-Irak2 or pc-DNA-control. The result showed that Irak2 was significantly upregulated in pcDNA-Irak2 group compared with matched control groups (control and pcDNA-control) (Fig. 7A,B). Afterwards, a CCK8 assay was executed and the data showed that overexpression of Irak2 reversed NEAT1 silencing-mediated repressive impact on cell viability (Fig. 7C,D). Simultaneously, enforced expression of Irak2 rescued NEAT1 silencing-mediated promoted effect on cell apoptosis (Fig. 7E). In-depth research demonstrated that the decreased levels of TNF-α, IL-6, IL-8 and IL-β in LPS+si-NEAT1 group were reversed following transfection with pcDNA-Irak2 (Fig. 7F,G). Taken together, these results suggest that upregulation of Irak2 transposed the NEAT1 silencing-mediated suppressive effect onto sepsis progression.

## DISCUSSION

Sepsis is a growing threat to humans and nearly 0.2 to 3 people per 1000 are affected by sepsis every year in the developed world (Jawad et al., 2012; Martin, 2012). lncRNAs have been confirmed to be associated with sepsis progression. Chen et al. found that upregulation of lncRNA UCA1 and HULC was required for pro-inflammatory response during LPS-induced sepsis in endothelial cells (Chen et al., 2019). Wang et al. reported that lncRNA SNHG16 modulated LPS-induced inflammatory pathways (Wang et al., 2018). Xu et al. found that lncRNA CRNDE correlated with sepsis-related inflammatory pathogenesis (Wang et al., 2019b). Lately, lncRNA NEAT1 was reported to function in sepsis-induced diseases. Zhang et al. confirmed that NEAT1 promoted inflammatory responses in sepsis-induced liver injury by the Let7a/TLR4 axis (Zhang and Niu, 2019). Yu et al. reported that NEAT1 alleviated sepsis-induced myocardial injury via the TLR2/NF-κB pathway (Wang et al., 2019a). In our research, NEAT1 was clearly upregulated in sepsis tissues and LPS-induced RAW 264.7 and HL-1 cells. In addition, downregulation of NEAT1 boosted viability and inhibited apoptosis of LPS-induced cells. We next checked the levels of inflammatory cytokines and found that knockdown of NEAT1 decreased the levels of TNF-α, IL-6, IL-8 and IL-β in LPS-induced cells. These results demonstrated that NEAT1 was involved in the regulation of sepsis progression.

Many reports confirm that lncRNAs could interact with miRNAs to modulate the sepsis progression (Wang et al., 2019b; Wang et al., 2018). In this study, miR-370-3p was confirmed to be the target of NEAT1, and the level of miR-370-3p was apparently downregulated in sepsis tissues and LPS-induced RAW 264.7 and HL-1 cells. Moreover, we also found that miR-370-3p was downregulated by NEAT1. Further investigation illustrated that the enhanced cell viability and declined apoptosis rate in LPS+si-NEAT1 group were reversed following transfection with anti-miR-370-3p. A previous study has shown that miR-370-3p was associated with inflammation injury in acute pneumonia (Zhang et al., 2019) and Tian et al. reported that upregulation of miR-370-3p inhibited inflammation cytokines including IL-6 and IL-1β (Hou et al., 2017). Similarly, our investigation showed that the decreased levels of TNF-α, IL-6, IL-8 and IL-β in LPS-induced RAW 264.7 and HL-1 cells were inverted after the infection with miR-370-3p inhibitor. In summary, our results demonstrated that NEAT1 could regulate sepsis progression by sponging miR-370-3p.

To deeply understand the regulatory mechanism of miR-370-3p in sepsis, we found its target gene, *Irak2*, which was correlated with LPS-induced inflammation injury (Guo et al., 2019). In this research, the mRNA and protein levels of Irak2 were clearly elevated in sepsis tissues and LPS-induced RAW 264.7 and HL-1 cells. Besides, NEAT1 regulated Irak2 expression by interacting with miR-370-3p. Further research demonstrated that overexpression of Irak2 transposed the repressive impact of NEAT1 silencing on sepsis progression. All in all, our results suggest that NEAT1 might serve as a sponge, interacting with and downregulating miR-370-3p, thus altering the expression of Irak2, eventually mediating the progression of sepsis in LPS-induced RAW 264.7 and HL-1 cells.

## Conclusion

In conclusion, our research demonstrated that NEAT1 silencing obstructed sepsis progression by decreasing the expression of Irak2 by sponging miR-370-3p. The NEAT1/miR-370-3p/Irak2 axis might contribute to improvements in the treatment of sepsis.

## MATERIALS AND METHODS

### Samples and cell culture

Tissues from sepsis patients and healthy volunteers were collected from Weifang People's Hospital, China. Informed consent was acquired from every participant and this research was authorized by the ethics committee of Weifang People's Hospital. The murine macrophage cell line (RAW 264.7) and murine cardiac muscle cell line (HL-1) were purchased from Sigma-Aldrich, (St Louis, MO, USA). McCoy's 5A medium (Sigma-Aldrich), containing 5% CO_2_ and 10% fetal bovine serum (FBS; Sigma-Aldrich) was used to culture cells. LPS (Solarbio, Beijing, China) was used to induce inflammation according to a previous report ([Bibr BIO049353C10][Bibr BIO049353C11]).

### Cell transfection

si-RNA against NEAT1 (si-NEAT1), miR-370-3p mimic (miR-370-3p) and miR-370-3p inhibitor (anti-miR-370-3p), as well as the corresponding controls (si-control, miR-NC, anti-miR-NC), were obtained from GenePharma (Shanghai, China). NEAT1 overexpression plasmid (pcDNA-NEAT1), Irak2 overexpression plasmid (named as pcDNA-Irak2) and matched control (pcDNA-control) were acquired from RiboBio (Guangzhou, China). Cell transfection was performed using Lipofectamine 3000 reagent (Invitrogen, Carlsbad, CA, USA) following the standard manufacturer's protocol.

### CCK-8 assay

RAW 264.7 and HL-1 cells, infected or not, were seeded into 96-well plates. Afterwards, the cells were treated with LPS for 1 h and then incubated with 10 μl CCK-8 solution (Beyotime, Shanghai, China) for 2 h. Optical density values were examined at 450 nm wavelength under the microplate reader (Bio-Rad, Richmond, VA, USA).

### RNA isolation and quantitative real-time polymerase chain reaction (qRT-PCR)

Total RNA was extracted using the TRIzol reagent (Beyotime). Then RNA was reversely transcribed to complementary DNA (cDNA) by PrimeScript™ RT Master Mix kit (Beyotime). The qRT-PCR was conducted by SYBR Green PCR Master Mix (Beyotime) and data were analyzed using 2^−ΔΔCt^ method. β-actin and U6 were introduced as the inner references. Primers used in this study:

NEAT1 (forward 5′-GTAATTTTCGCTCGGCCTGG-3′, reverse 5′-TACCCGAGACTACTTCCCCA-3′); miR-370-3p (forward, 5′-GCCTGCTGGGGTGGAACCTGGT-3′, reverse 5′-CTCAACTGGTGTCGTGGA-3′); Irak2 (forward, 5′-CATGGCTTGCTACATCTACC-3′, reverse 5′-ACGTTTGTCTGTCCAGTTGA-3′); β-actin (forward 5′-GCACCACACCTTCTACAATG-3′, reverse, 5′-TGCTTGCTGATCCACATCTG-3′); U6 (forward, 5′-TCCGGGTGATGCTTTTCCTAG-3′, reverse, 5′-CGCTTCACGAATTTGCGTGTCAT-3′).

### Flow cytometry

An Annexin Apoptosis Detection Kit (Sigma-Aldrich) was used to check cell apoptosis following the given procedures. Briefly, cells were resuspended using the binding buffer and then 5 μl Annexin V-fluorescein isothiocyanate and 5 μl Propidium Iodide were added to the buffer to incubate for 5 min in the dark. The stained cells were analyzed by flow cytometry (Countstar, Shanghai, China).

### Cytokines measurement

The concentrations of TNF-α, IL-6, IL-8 and IL-β were determined using the corresponding enzyme linked immunosorbent assay (ELISA) kits (Beyotime). Briefly, RAW 264.7 and HL-1 cells were seeded in 96-well plates and were treated with LPS. The supernatant of cell culture was used for determination of cytokine concentration by ELISA according to the manufacturer's protocol.

### Dual-luciferase reporter assay

The potential complementary sequences between miR-370-3p and NEAT1 or Irak2 were forecasted by starBase (Li et al., 2014). The dual-luciferase reporter assay was performed according to a previous paper (Li et al., 2016). The wild-type sequence of NEAT1 or Irak2 3′UTR harboring the binding sites of miR-370-3p was inserted into the pGL3 vector (Promega, Madison, WI, USA) to construct the luciferase reporter vector WT-NEAT1 or Irak2 3′UTR-WT, respectively. Similarly, MUT-NEAT1 and Irak2 3′UTR-MUT reporter vectors were established by mutating the potential target sites of miR-370-3p. Then, the vectors with miR-370-3p or miR-NC were cotransfected into RAW 264.7 and HL-1 cells using Lipofectamine 3000 (Invitrogen). The Dual-Glo^®^ Luciferase Assay System kit (Promega) was used to measure luciferase activity.

### Western blot

Proteins from samples were isolated using RIPA buffer (Vazyme, Nanjing, China) and were segregated by sodium dodecyl sulfate polyacrylamide gel electrophoresis, and then proteins were transferred onto the polyvinylidene diﬂuoride membranes (Vazyme). The membranes were blocked with 5% skimmed milk (Vazyme). Thereafter, the membranes were incubated with the primary antibodies: anti-Irak2 (1:2000, ab62419, Abcam, Cambridge, UK) or glyceraldehyde 3-phosphate dehydrogenase (1:2500, ab9485, Abcam) overnight. After being rewashed, the membranes were incubated with the secondary antibody (1:3000, ab205718, Abcam) for 2 h. The membranes were analyzed by the ChemiDoc™ MP Imaging System (Bio-Rad) after being treated with ECL kit (Vazyme).

### Statistical analysis

Experimental data were calculated by GraphPad Prism (GraphPad, La Jolla, CA, USA) and presented by mean±standard deviation (s.d). Two independent groups were compared by using a Student's *t*-test. For more than two groups, the one-way analysis of variance (ANOVA) was used to assess the difference. Pearson's correlation coefficient was applied to analyze the correlation between NEAT1 and miR-370-3p or Irak2 in sepsis tissues. Every experiment was repeated at least three times independently. *P*<0.05 represented statistical significance.
